# Carbohydrate inhibitors of cholera toxin

**DOI:** 10.3762/bjoc.14.34

**Published:** 2018-02-21

**Authors:** Vajinder Kumar, W Bruce Turnbull

**Affiliations:** 1 Department of Chemistry, Akal University, Talwandi Sabo, Punjab, India; 2 School of Chemistry and Astbury Centre for Structural Molecular Biology, University of Leeds, LS2 9JT, UK

**Keywords:** carbohydrate, cholera, multivalency, toxin

## Abstract

Cholera is a diarrheal disease caused by a protein toxin released by *Vibrio cholera* in the host’s intestine. The toxin enters intestinal epithelial cells after binding to specific carbohydrates on the cell surface. Over recent years, considerable effort has been invested in developing inhibitors of toxin adhesion that mimic the carbohydrate ligand, with particular emphasis on exploiting the multivalency of the toxin to enhance activity. In this review we introduce the structural features of the toxin that have guided the design of diverse inhibitors and summarise recent developments in the field.

## Introduction

Cholera, meaning a flow of bile, is caused by an acute enteric infection of the Gram-negative facultative anaerobe *Vibrio cholerae*. Not only does this disease have a disastrous effect on health, it also impacts on the socioeconomic status of societies where it is endemic. The *V. cholerae* bacterium was identified by Robert Koch in 1883, and ever since then, this scourge has grown continuously with catastrophic effects on millions of people [1]. Although appropriate water, hygiene and sanitation interventions can reduce incidence of bacterial infection, the WHO predicts that there will still continue to be millions of deaths due to diarrhoea in the developing nations of the world. While cholera is rare and seldom life threatening in developed countries, it can still pose a risk to those at the extremes of age and the immunosuppressed. However, Hispaniola Island and western African countries (Ghana, Guinea, Guinea-Bissau, Niger and Sierra Leone) are completely under the control of this epidemic. According to annual statistics of 2016 in the *Weekly Epidemiological Record* (WER) by the WHO, 172454 cases are reported in 42 endemic countries including 1304 deaths. Among 42 countries, Afghanistan, the Democratic Republic of the Congo (DRC), Haiti, Kenya, and the United Republic of Tanzania were majorly affected [2]. Recent data for the year 2017 from the GIDEON internet site (that continuously scans Medline, WHO, CDC and other peer reviewed journals), highlights the recent cholera outbreak principally affecting Somalia, DRC and Tanzania [3]. The total number of cases reported in these countries was almost 65,000 leading to 1500 deaths so far. In the Americas, the Haiti region has been fighting this epidemic since October 2010. As of June 2017, the outbreak was still ongoing and a total of over 800,000 cases, including 10,000 deaths, had been registered [3]. This infection also prevails in the Dominican Republic and Cuba [2]. Furthermore, deaths due to cholera in Asian countries constitute 3% of the world’s total [2]. However, this may be underestimated as limitations in surveillance systems in large parts of Asia, lead to millions of cholera cases not being recorded. After broad analysis, Ali et al. estimated that 2.9 million cases and 95,000 deaths happen every year worldwide [4]. Thus cholera continues to be a serious concern in many parts of the globe.

The agent responsible for causing diarrhea is an AB_5_ toxin released by the bacteria. Thus, an understanding of this toxin becomes essential in finding/developing molecules that could prevent cell entry of the toxin and inhibit its activity. AB_5_ toxins are an important class of bacterial toxins. They consist of a single A-subunit and a pentamer of B-subunits [5]. The catalytic activity of the toxins is due to the A-subunit, while the B-subunit enables binding of the complex to the cell surface and its delivery into the target cells, hence the complete AB_5_ holotoxin is required for their toxic effects. Because of the difference in the sequence homology and catalytic activity, the classes of AB_5_ toxins are subdivided into three families (Figure 3): the cholera toxin (CT) family, the shiga toxin (ST) family and the pertussis toxin (PT) family [6]. The CT family contains CT, and heat-labile toxins LT-I and LT-II [7–8]. The ST family contains the shiga toxins (SHT) themselves and the related verotoxins (also known as shiga-like toxins: SLT-I, SLT-II) [9–10] and SHT toxin comes from *Shigella dysenteriae* and verotoxin comes from enteropathogenic *E. coli* strains such as O157-H7. SHT and SLT-I are almost identical, with very little difference in the A-subunit. But the SLT-II shows more deviation in its gene sequence from the SHT and SLT-I toxins [9]. Sequence homology in the CT family is high between CTB and LTI-B (80% identical), but much lower between these proteins and the LTIIa and LTIIB toxins. PT is quite unusual in that all five of its B-subunits are different, but overall, an AB_5_ architecture is still preserved [11]. A detailed knowledge of the 3D structure of these toxins is informative for the design of effective inhibitors.

## Review

### Structure and function of cholera toxin

Many crystallographic studies of the AB_5_ toxins have been undertaken over the past 20 years [8–14]. Here, we focus solely on those describing the structure of the cholera toxin.

#### A-Subunit

The A-subunit of CT is the catalytic site of the AB_5_ toxin, and forms a complex with the B-pentamer [15]. It is initially expressed as a single polypeptide chain which is cleaved by a protease to give two subunits, A1 and A2, remain held together by extensive non-covalent forces and a single interchain disulfide bond [16]. The A2-subunit acts as a linker between the toxic A1-subunit and CTB which is the delivery vehicle that can transport the complex into cells and direct the toxin to the endoplasmic reticulum, from where it can escape into the cytosol. The A1 chain has ADP-ribosyltransferase activity that allows the toxin to covalently modify the α-subunit of the stimulatory G protein G_sα_ so that it remains in its active GTP-bound state. The consequence of this change is to produce high levels of cAMP which activates protein kinase A to phosphorylate the cystic fibrosis transmembrane conductance regulator which is a chloride ion channel [15]. Transport of chloride ions to the intestine is accompanied by excessive amounts of water entering the gut and the diarrhea that is symptomatic of cholera.

**Figure 1:**
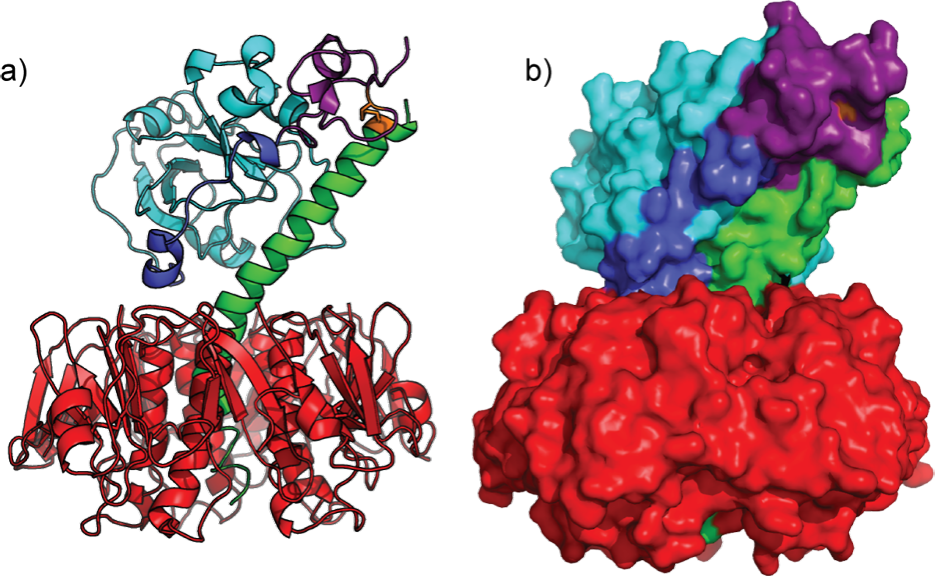
a) Ribbon and b) surface depictions of the cholera toxin: A1_1_ domain in light blue; A1_2_ domain in dark blue; A1_3_ domain in purple; cystine disulfide in orange; A2 peptide in green and B-subunit in red. Figure prepared using the PyMOL programme from Protein Data Bank file 1XTC.pdb.

The A1-subunit consists of three domains namely A1_1_, A1_2_ and A1_3_ (Figure 1). While the A1_1_ domain is responsible for catalysis, the A1_2_ and A1_3_ domains have been implicated in allowing the A1 subunit to escape from the endoplasmic reticulum (ER) into the cytosol. Following arrival in the ER, protein disulfide bond isomerase can reduce the disulfide bond between A1_3_ and A2, releasing the A1 protein and causing the A1_2_ and A1_3_ domains to unfold [17]. The protein is then recognised by the cell as a misfolded protein and is exported into the cytolsol for degradation. However, once in the cytosol, it binds to another protein Arf6, which stabilizes the A1_2_/A1_3_ domains and activates the A1 enzyme.

#### B-Subunit

**Figure 2:**
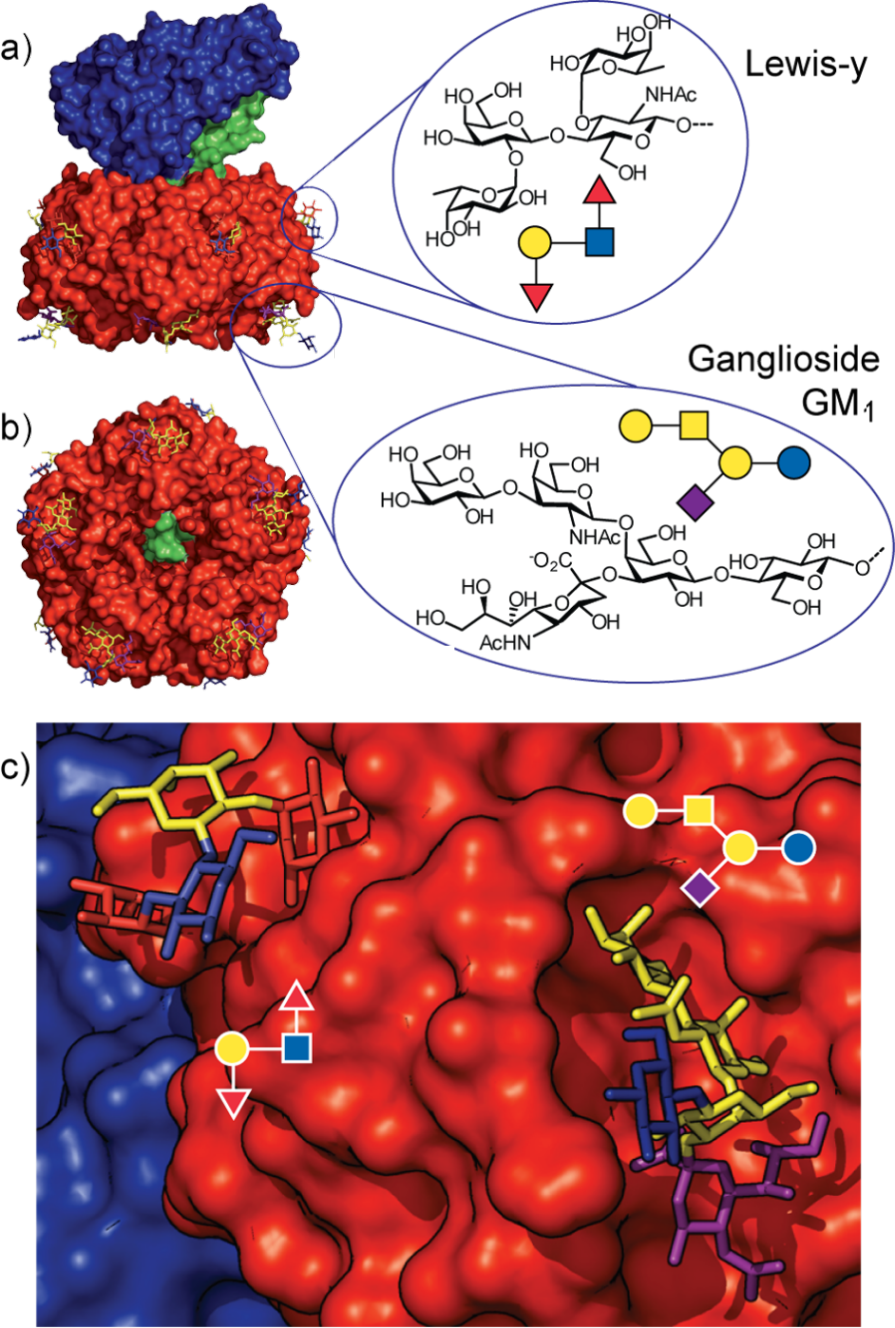
a) Structure of the cholera toxin showing the location of its carbohydrate binding sites and the structures of the Lewis-y and Ganglioside GM_1_ ligands; A-subunit (blue), B-subunit (red) and the A2 peptide linker (green). b) Bottom face of the toxin showing the symmetry of the B-subunit and the A2 peptide linker emerging through the central channel. c) Close-up view of the two sugar binding sites. Figure prepared using the PyMOL programme from Protein Data Bank files 1XTC.pdb, 3CHB.pdb and 3EFX.pdb.

The B-subunit (CTB) is a homopentamer [18–19], and crystallographic data on B subunits of the CT family showed very little deviation (less than 0.5 r.m.s.) from exact rotational symmetry. Five long α-helices surround the central cylindrical pore through which the A2-subunit is threaded. Each subunit of a B-pentamer has a single binding site for the GM_1_ oligosaccharide on the face of the pentamer distal to the A1-subunit [12,14]. GM_1_ is a branched pentasaccharide [Galβ1-3GalNAcβ1-4(NeuAcα2-3)Galβ1-Glcβ1-1-ceramide] bearing a ceramide moiety at the anomeric center of the Glc moiety (Figure 2). The terminal galactose residue of GM_1_ is buried most deeply inside the cavity of CTB [12,14], while the sialic acid branch sits in a wider shallow pocket. Both of these terminal sugar residues show hydrogen bonding interactions with the protein and associated water molecules. The GM_1_ oligosaccharide (GM_1_os) binds very tightly to CTB with a dissociation constant (*K*_d_) of around 40 nM (measured by isothermal titration calorimetry, ITC), while simple galactosides have millimolar *K*_d_s and little interaction can be detected for simple sialosides [20]. The distance separating the binding sites is similar for all members of the AB_5_ toxin family and is believed to be instrumental in clustering the glycolipid ligands in such a way that membrane curvature is induced upon binding [21].

More recently, a second binding site has been discovered on the edge of the B-subunit sitting closer to the A-subunit face (Figure 2) [13,22–25], This secondary binding site recognises fucosylated structures including blood group oligosaccharides of the Lewis-y family. Individually, the interactions are much weaker than the CTB-GM_1_os interaction (*K*_d_ ca. 1 mM measured by ITC), but even these weak binding interactions can still be functionally useful once the effect of multivalent binding enhancement has been taken into consideration. Indeed, ITC experiments have also shown the highest affinity site on the SLT-1 B-subunit has a *K*_d_ of only 1 mM [26], yet the toxin achieves sub-nanomolar affinity at a cell membrane. The purpose of the CTB blood group oligosaccharide binding site remains a topic for debate, but it may be responsible for the reported blood group dependence of the severity of cholera [13,24,27], or it could provide an independent route for cell entry through interactions with cell surface glycoproteins [28].

### Structure-based design of inhibitors for cholera toxin

The availability of crystal structures for cholera and *E. coli* heat-labile toxins has driven opportunities for the design of potent inhibitors for these toxins. While some interest has been shown in the possibility of inhibition of cholera toxin assembly and inhibition of the enzymatic activity, most effort has been invested in seeking inhibitors of the adhesion process [29].

Designing the inhibitors for the receptor-binding process is a very compelling strategy, because the inhibitors would fight the toxin in the intestinal tract of the human host. Therefore ligands need not to cross any barrier and there is no constraint on ligand size. In the past years, several strategies have been drawn for the receptor binding to AB_5_ toxins; while some target on the individual binding sites, others are intended at designing multivalent ligands against the entire toxin B pentamer [6,30–31].

### Monovalent receptor-binding inhibitors

**Figure 3:**
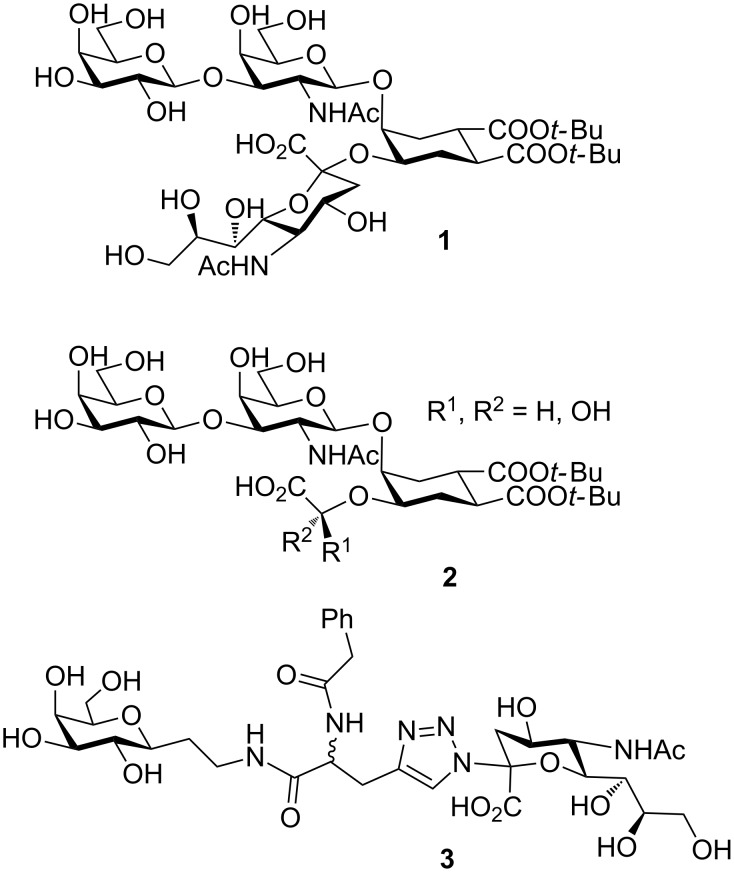
Bernardi and co-workers’ designed oligosaccharide mimetics of GM_1_.

Bernardi and co-workers designed carbohydrate derivatives that mimic the natural CT receptor, ganglioside GM_1_ [32]. They replaced the central 3,4-disubstituted Gal unit of GM_1_ with dicarboxy cyclohexanediol (DCCHD, Figure 3). DCCHD exhibits the same absolute and relative configuration of the natural galactose residue. Taking this into account, a pseudo-tetrasaccharide **1** was made in which the recognition units, the terminal galactose and Neu5Ac, were attached onto the DCCHD scaffold. Inhibition assays of the oligosaccharide mimetic with CT and LT showed similar potency as that of natural ligands [32]. But, the alpha-sialylation was the bottleneck step in the synthesis, so they designed second generation inhibitors by changing the synthetically challenging α-Neu5Ac with alpha-hydroxy acids **2** [33–34]. Using a combinatorial approach, a library of non-hydrolyzable, non O-glycosidic third generation inhibitors were synthesised using appropriate linkers. The CTB affinity of these inhibitors was measured using weak affinity chromatography and some molecules displayed enhancement of affinity over the individual epitome ‘Galactose’ [35]. One such compound **3** has found to co-crystallise with CTB in a way that the galactose and sialic acid groups bind to adjacent CTB pentamers in the crystal lattice, opening a possible route for the structure-based design of inhibitors that aggregate the toxin [36].

**Figure 4:**
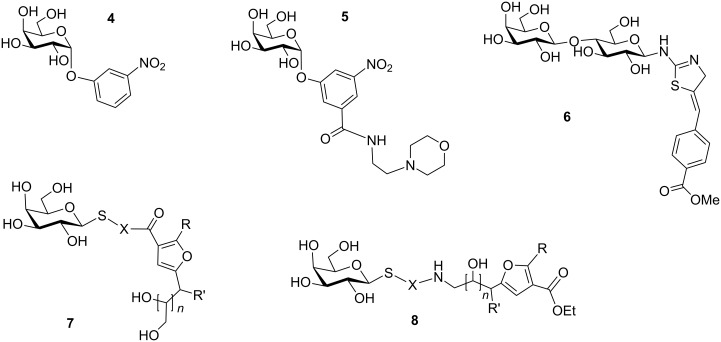
Structure of monomeric ligands. X = amino acid residues, aminoalkyl, 1,2,3 triazoles; *n* = 1, 2; R = H, Me, R' = OH, NHAc.

Hol, Verlinde and co-workers designed and synthesised a library of compounds utilizing a fragment of the toxin’s natural receptor. Both CTB and LTB have specific affinity for the terminal galactose part of GM_1_ [37–39]. They screened a number of galactose derivatives with substitution at O1 and C2 and found that the most potent molecule in this library was *m*-nitrophenyl α-D-galactoside (**4**) which was 100 times better than galactose for binding to CTB [38–39]. In another report, Mitchell et al. designed and synthesised twenty 3,5-substituted phenylgalactosides, e.g., **5** and when these compounds were tested on CT it was found that they have a six-fold higher affinity than *m*-nitrophenyl α-D-galactopyranoside (Figure 4) [40].

Vrasidas et al. synthesised a simple lactose-2-aminothiazoline conjugate as a CT antagonist. Its affinity for CTB was determined by monitoring the change in fluorescence of tryptophan-88, located in the GM_1_ binding site, upon titration of the protein with the inhibitor. Compound **6** showed excellent binding with a *K*_d_ value of 23 µM [41]. Robina and co-workers synthesised non-hydrolyzable S-galactosides and non-carbohydrate ligands based on polyhydroxyalkylfuronate moieties and measured their affinities by weak affinity chromatography (WAC) and also studied their interaction by saturation transfer difference NMR experiments [42]. Although, these compounds, **7** and **8**, did not display good inhibition, the non-glycosylated ligands offered new avenues for better CT ligand designs.

### Multivalent receptor-binding inhibitors

The five-fold symmetry of AB_5_ toxins provides a strong encouragement to think about multivalent inhibitor design from (even weakly binding) monovalent inhibitors [30–31]. Multivalent ligands have been long applied to a wide range of protein targets [43–45]. By having an inhibitor that may bind simultaneously with multiple binding sites, the dissociation rate of the complex is effectively reduced. Even if any individual ligand group dissociates from the protein, then the others will continue to make contact between the protein and the inhibitor, thus maintaining a high effective concentration of the dissociated ligand group in the vicinity of the binding site and increasing the probability of rebinding occurring. The gain in inhibitory potency for the multivalent ligands can be in many orders of magnitude. Here we have divided multivalent ligands and inhibitors of cholera toxin into three classes: sub-pentavalent inhibitors; pentavalent inhibitors; and inhibitors containing more than five ligands.

#### Sub-pentavalent inhibitors

**Figure 5:**
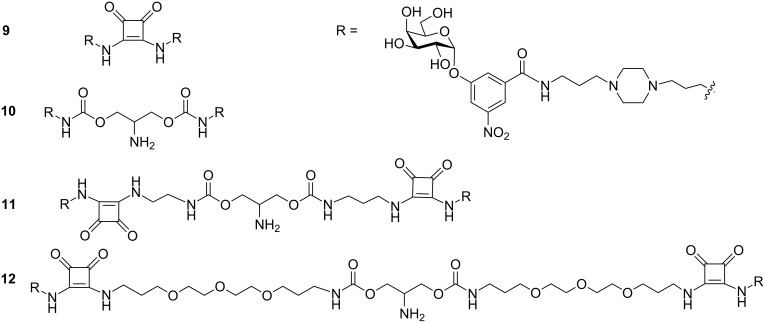
Bivalent inhibitor designed and synthesised by Pickens et al.

Hol and Fan [46] designed and synthesised both spanning and non-spanning bivalent inhibitors. “Spanning” means the ligand has sufficient length of the linker to reach the two binding moieties of CT, whereas “non-spanning” means there is insufficient linker length for intra-pentamer chelation, but the second galactosyl moiety could bind to another CT molecule. They found that non-spanning bivalent inhibitors **9**–**12** as shown in Figure 5, show more binding affinity than the monovalent ones, which could also be derived from a statistical effect of a higher rebinding rate.

**Figure 6:**
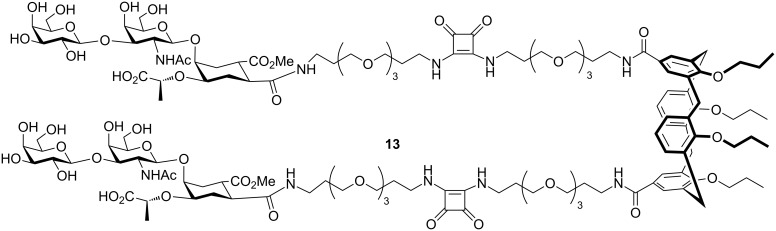
Bivalent inhibitor designed and synthesized by Arosio et al.

Bernardi, Casnati and co-workers prepared a bivalent ligand **13** for CT by attaching two copies of GM_1_ mimic compound **3** to a calixarene (Figure 6) [47]. By measuring the affinity for CT by fluorescence titration, they found that the enhancement in affinity was 3800-fold as compared to the GM_1_ mimic, which is consistent with a chelating mechanism.

**Figure 7:**
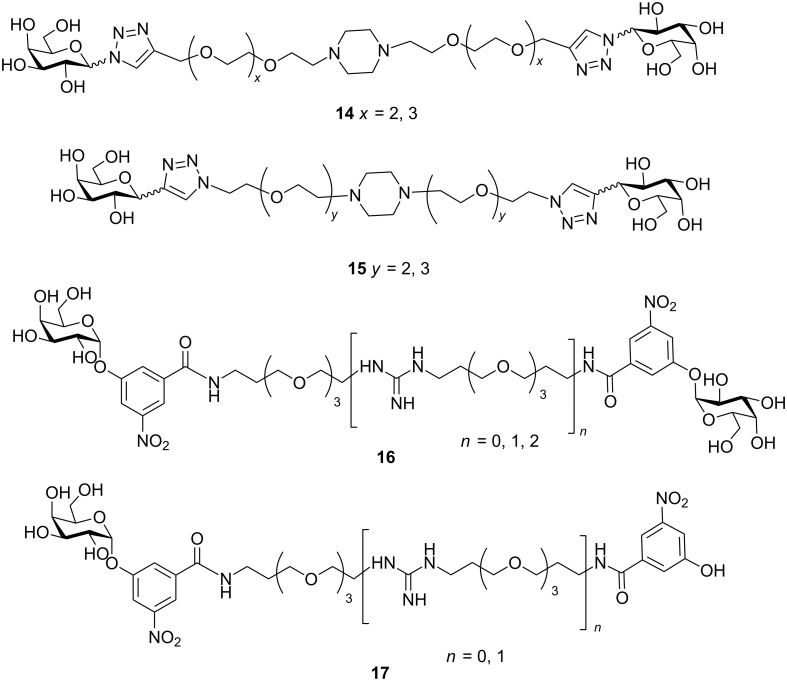
Bivalent inhibitors designed and synthesised by Leaver and Liu.

Hughes and co-workers synthesised and evaluated bivalent 1,2,3 triazole-linked galactopyranosides **14** and **15** as shown in Figure 7 [48]. They used a piperazine core as central divalent core on to which the galactose units were attached via flexible linkers. They found that these compounds exhibit binding affinity one order higher than *m*-nitrophenyl galactopyranoside (**4**) [48]. In another recent report, low molecular weight poly(*N*-acryloylmorpholine) was used to link galactose residues to form a bivalent inhibitor, but the biological assay demonstrated only moderate inhibitory activity [49]. Liu et al. synthesised bivalent ligands **16** and **17**, for evaluation through biophysical techniques (Figure 7) [50]. They found that the enhancement in affinity and potency was due to non-specific interactions between the linker portion, nitrophenyl group and CT. The interactions increase as linker length increase. Hence, they concluded that the length, size and chemical nature of the ligand has a major effect on binding with the protein toxin.

**Figure 8:**
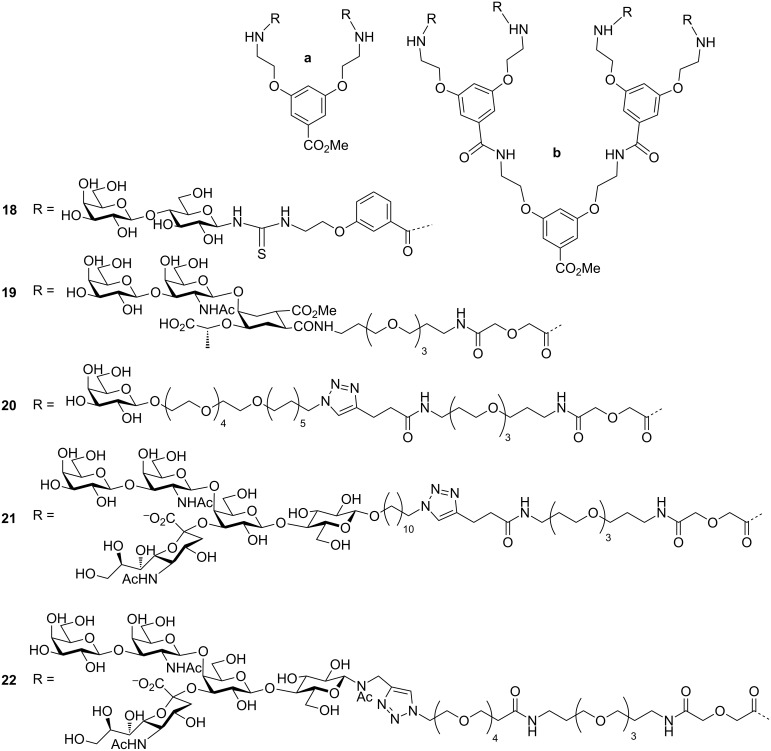
Bivalent and tetravalent inhibitor designed and synthesised by Pieters, and Bernardi et al.

While the ganglioside GM_1_ head group is the highest affinity natural ligand for CTB, galactose and lactose (Figure 8) head groups have also been used for synthesising bivalent and tetravalent multivalent inhibitors and showed substantial gains in binding affinity in comparison to the corresponding monovalent ligands. Pieters and co-workers attached a lactose-derived monomeric ligand to the dendrimer **18**, and found that there was an affinity and potency gain from divalent and tetravalent molecules [51]. Even the galactose containing dendrimers **20** bind as strongly as that of GM_1_ [52]. As an improved design of ligand for CT, the GM_1_ mimic synthesised by Bernardi and co-workers was attached to the dendrimer synthesised by the Pieters group and hence compounds **19** and **21** were obtained [53]. The divalent compound **19a** and tetravalent compound **19b** exhibited IC_50_ values of 13 and 0.5 µM, respectively. In another report, they reported that the divalent compound **21a** and tetravalent compound **21b** displayed 9,500 and 83,000-fold enhanced potency, respectively, than monovalent GM_1_ [54–55].

Fu et al. synthesised a tetravalent ligand containing highly hydrophilic spacer arms **22b**, and found that this ligand demonstrated almost the same potency with an IC_50_ value of 160 pM as that of **21b** (IC_50_ = 190 pM) [56].

**Figure 9:**
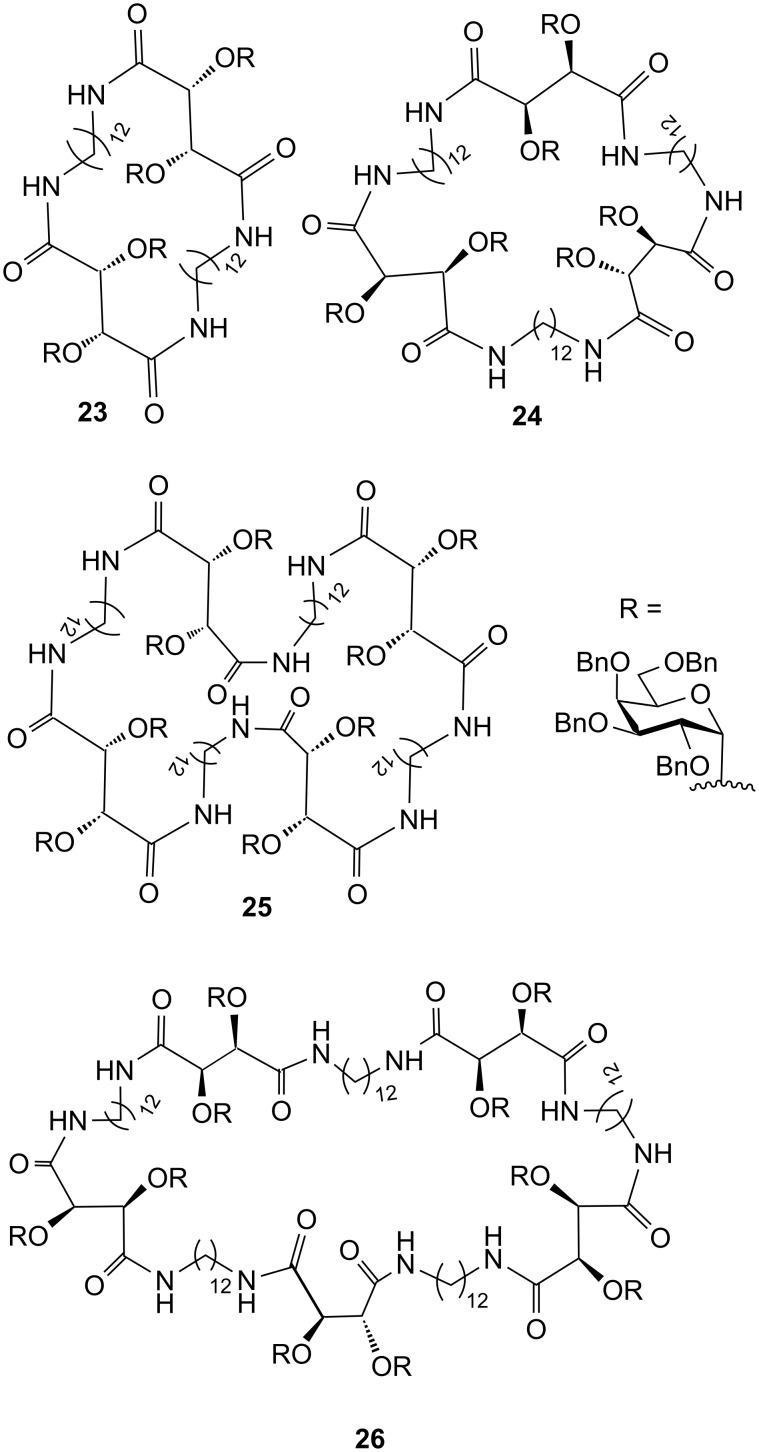
Cyclic inhibitors synthesised by Kumar et al. for CT.

To reduce the energy loss in the form of entropic penalty to be paid on binding, Kumar et al. synthesised noncyclic and cyclic neoglycopeptides and glycoamides for cholera toxin, e.g., **23**–**26** (Figure 9) [57]. They prepared divalent, trivalent, tetravalent, cyclic divalent, cyclic trivalent, cyclic tetravalent and cyclic pentavalent inhibitors with large cyclic core structures.

#### Pentavalent inhibitors

**Figure 10:**
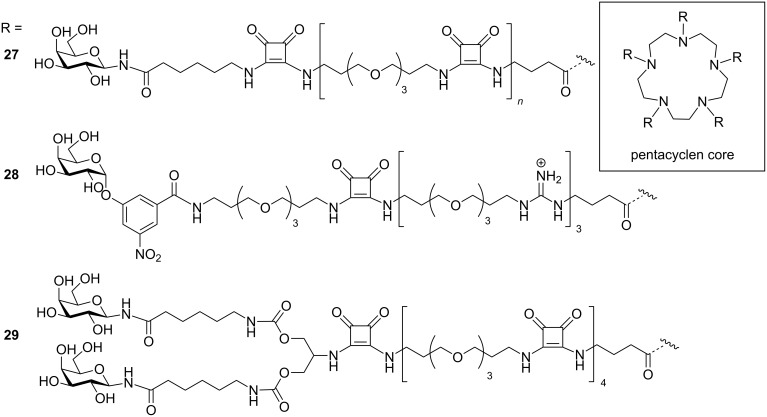
The star-shaped inhibitors reported by Fan, Hol and co-workers.

The pentameric structure of CTB has proved to be an enticing invitation to many scientists to develop multivalent inhibitors that are also pentavalent. Fan, Hol and co-workers were first to design and synthesise pentavalent inhibitors **27**–**29**, for the LTB/CTB [58] (although Bundle and co-workers were also working on analogous designs for shiga-like toxin [59]). They synthesised the inhibitors on a pentacyclene core on which galactose and *m*-nitrophenyl-α-D-galactopyranoside were attached by long flexible linkers (Figure 10) [60–61]. They found million-fold increases in activity in comparison to the corresponding monovalent inhibitors with IC_50_ values of 40 nM.

**Figure 11:**
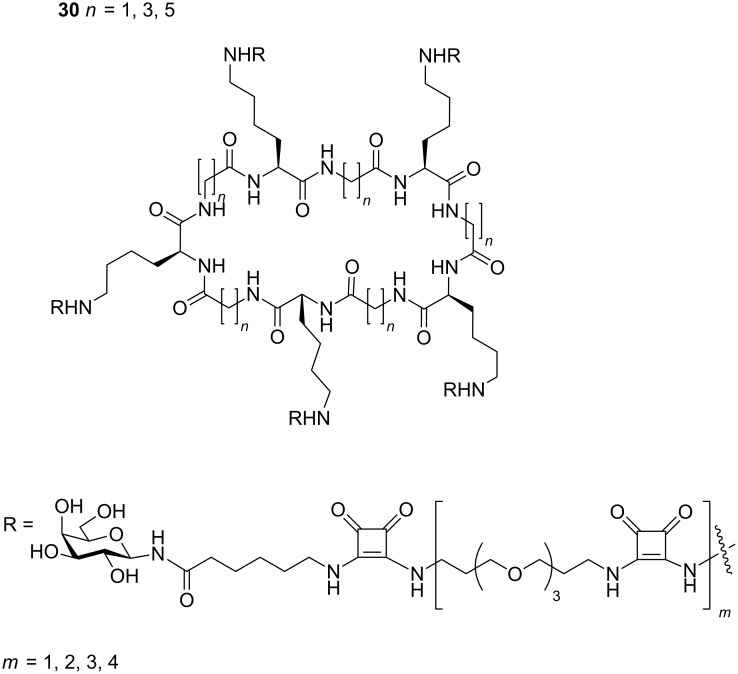
Differently sized cyclic decavalent peptide core designed by Zhang et al.

Zhang et al. synthesised large cyclic decapeptides (up to 50 atoms in the ring) in a “core-linker-finger” modular setup (Figure 11) [62]. These compounds **30** showed good inhibitory results with IC_50_ values 100,000-fold more potent than monovalent galactose. This strategy facilitated a methodical study to measure the effect of linker length on the affinity of the pentavalent ligands towards the target toxin. Large affinity-gains were achieved for pentavalent ligands with short linkers on these large cyclic cores, indicating that the central cyclic peptide core probably has an expanded ring conformation.

**Figure 12:**
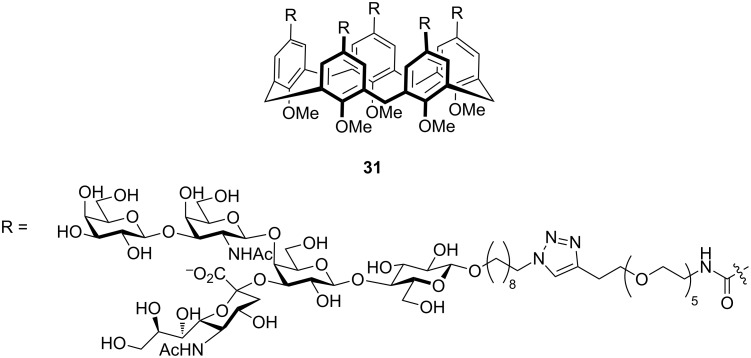
Calix[5]arene core-based pentavalent inhibitor designed by Garcia-Hartjes et al.

Garcia-Hartjes et al. synthesised and evaluated the GM_1_os linked calix[5]arene molecule **31** as shown in Figure 12, and found that compound **31** displayed 100,000 times more potency as compared to GM1os derivatives having an IC_50_ value of 450 pM [63].

**Figure 13:**
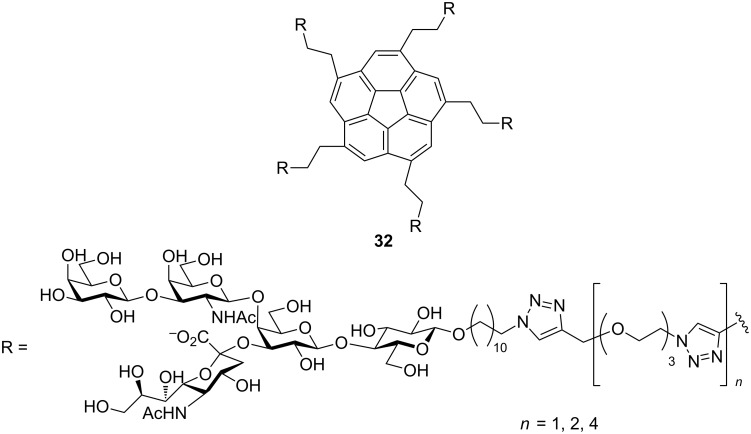
Corannulene core-based pentavalent inhibitor designed by Mattarella et al.

In another report Siegel and co-workers showed that corannulene-based pentavalent glycocluster **32** (Figure 13) bearing GM_1_os moieties possessed affinity for CT in low nanomolar range [64]. The IC_50_ value obtained was in the range of 5–25 nM.

**Figure 14:**
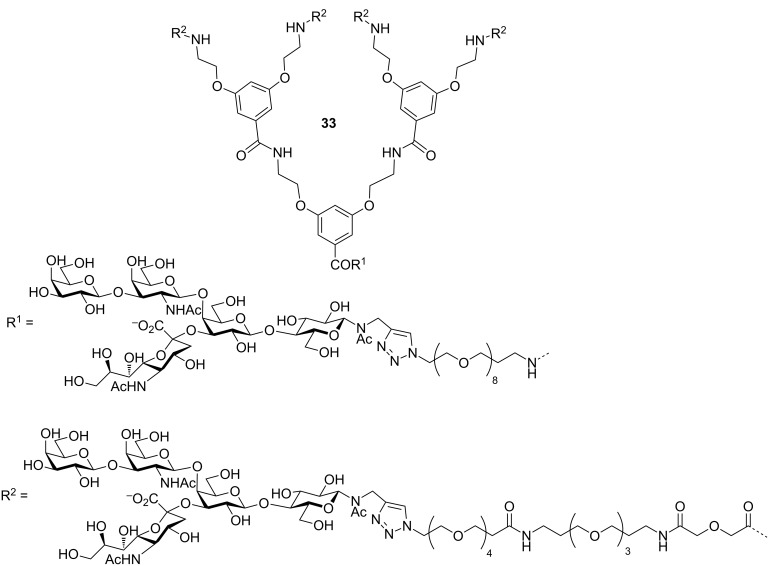
Pentavalent inhibitor designed by Pieters and co-workers.

Fu et al. also synthesised and evaluated a pentavalent inhibitor **33** (Figure 14), analogous to their tetravalent compound **22b** to investigate the difference between matching or mismatching the valency with that of the target CTB protein [56]. Previous biophysical studies had suggested that a mismatch in the valencies of ligand and receptor favoured an aggregation mechanism for inhibition [55] whereas matching the valency has previously been assumed to lead to the formation of 1:1 complexes [59–64]. They found that the potency exhibited by compound **33** in the usual enzyme-linked lectin assay (IC_50_ = 260 ± 20 pM) was slightly lower that for the tetravalent compound **22b** (IC_50_ = 160 ± 40 pM) [56]. Inhibition results described in this review are essentially all derived from very similar types of enzyme-linked lectin assays (ELLA) in which the inhibitors are used to prevent CTB-linked horseradish peroxidase from binding to microtitre plates coated with the ganglioside GM_1_ ligand. However, it is important to note that IC_50_ values are always dependent on the experimental design and the potency of some compounds may be underestimated if the concentration of the target protein is similar to or higher than the measured IC_50_ value. Pieters and co-workers have recently reported a new type of inhibition assay based on cultured intestinal organoids [64], which when treated with the CT holotoxin swell up as fluid is transported across their epithelia. Toxin inhibition is quantified by measuring the reduction in organoid swelling. When inhibitors **22b** and **33** were re-evaluated using this new assay, they were found to be even more active than previously measured in the ELLA (IC_50_ = 34 pM for **22b** in organoid assay vs 160 pM in ELLA; IC_50_ = 15 pM for **33** in organoid assay vs 260 pM in ELLA). While enzyme-linked lectin assays will undoubtedly continue to be a popular method for easily evaluating and comparing different inhibitors, the intestinal organoid assay introduced by Pieters and co-workers is now the most sensitive and realistic in vitro assay available [65].

**Figure 15:**
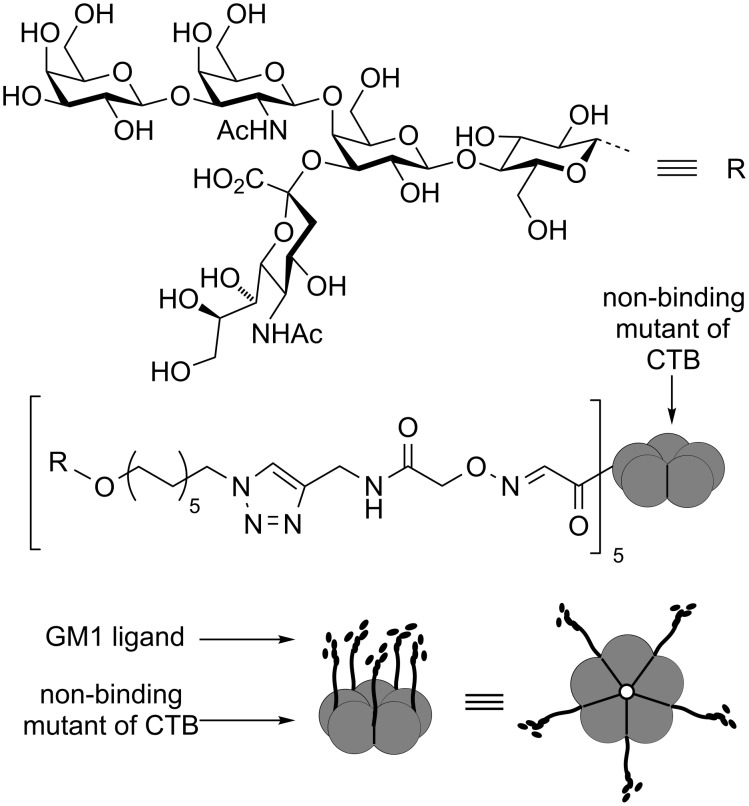
Neoglycoprotein inhibitor based on a non-binding mutant of CTB.

Branson et al. took a different approach to scaffold design in which they made a non-binding mutant of the target CTB protein [66], oxidised the N-terminal threonine residue of each subunit to an aldehyde and then chemically attached GM_1_os ligands by oxime ligation (Figure 15). This neoglycoprotein was able to display the five copies of the carbohydrate ligand with appropriate spacing’s to maximize interactions with the target protein. Dynamic light scattering and analytical ultracentrifugation demonstrated that the glycoprotein formed a 1:1 complex with the target CTB protein and was highly effective as an inhibitor with an IC_50_ value of 104 pM.

#### Multivalent inhibitors with more than five ligands

**Figure 16:**
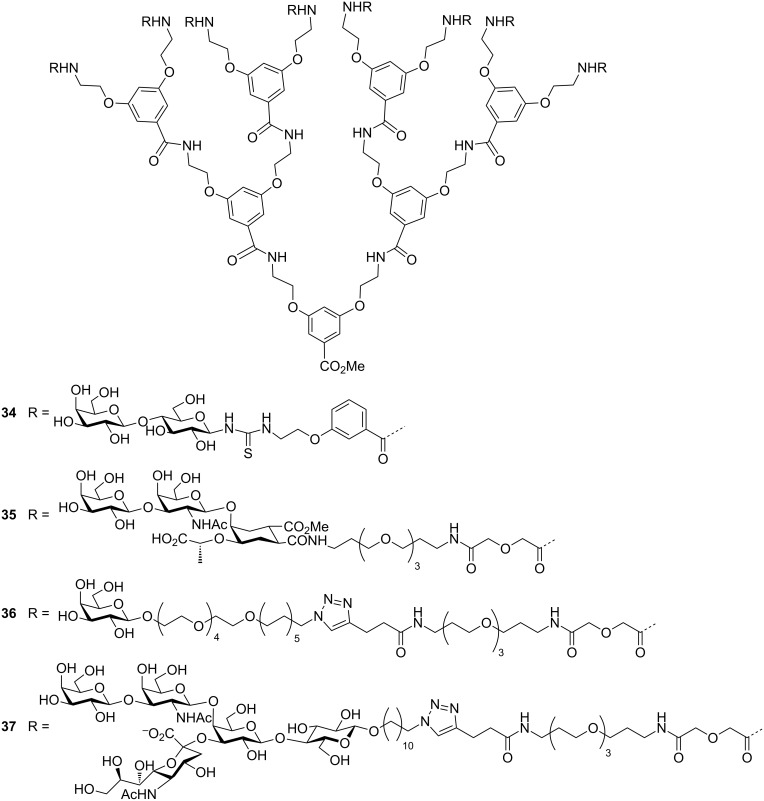
Octavalent inhibitor designed by Pieters, Bernardi and co-workers.

While pentavalent inhibitors are seductive as they match the symmetry with the CTB protein, many researchers have sought to exceed the valency of five. In many cases this is largely for convenience of preparation of polymers bearing multiple pendant groups, or to achieve inhibitors that are sufficiently long to cross-link the binding sites in a protein. Also, if multivalent molecules have not been specifically designed to match the distance between the target binding sites, then sometimes larger multivalent compounds are better. For example, Pieters and co-workers used a tryptophan fluorescence quenching assay to show that octavalent lactose-based dendrimer **34** (Figure 16) had a *K*_d_ value of 33 µM as compared to monovalent lactose derivative having a *K*_d_ value of 18,000 µM [51]. Hence, compound **34** displayed 545 fold more potency per lactose unit than monovalent lactose. In another report, they found that octavalent galactose-derived dendrimer **36** displayed excellent CT inhibition with an IC_50_ value of 12 µM and this was better than monovalent GM_1_os (IC_50_ = 19 µM) [52]. From the collaboration work of Pieters and Bernardi, an ELISA assay confirmed that compound **35** (Figure 16) was the most potent compound having an IC_50_ value less than 0.5 µM [53]. In another report, they reported that the octavalent GM_1_os dendrimer complex **37** (Figure 16) displayed a 380,000-fold enhanced potency relative to monovalent GM_1_ [54].

**Figure 17:**
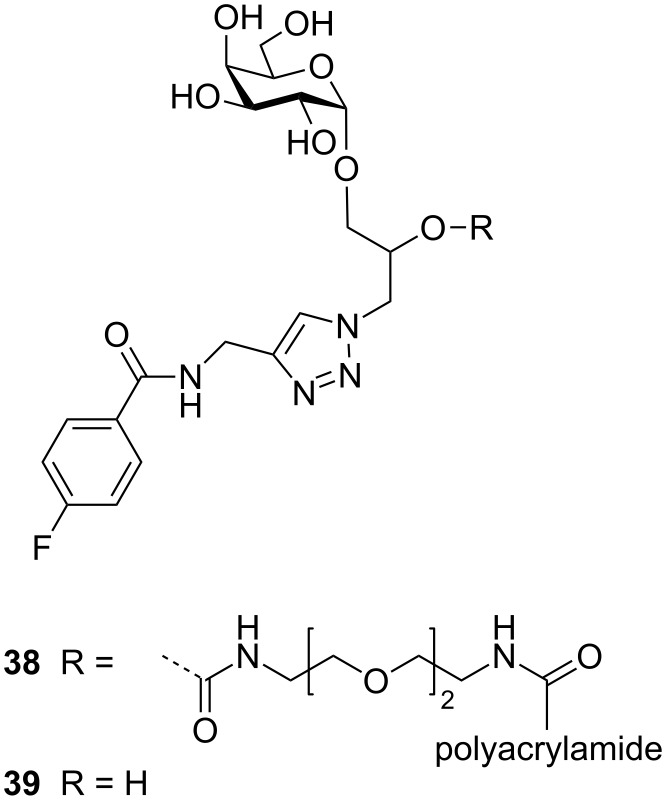
Hetero-bifunctional inhibitor designed by Bundle and co-workers.

Polymeric scaffolds have also been used extensively over many years [67]. Some recent highlights have included using polymer backbones to identify GM1 analogues that can give enhanced multivalent interactions [68], evolving glycopolymers using exchangeable ligands [69], and tuning the way the ligands are connected to the polymer backbone for maximum interaction [70–71]. For example, using a fragment-based approach, Tran et al. synthesised and evaluated a library of polymer-based hetero-bifunctional ligands and found that some compounds showed low nanomolar multivalent inhibition [68]. Alpha-galactoside **38** (Figure 17) showed the highest activity when presented on the polymer scaffold with an IC_50_ value of 0.005 µM. In contrast, the IC_50_ value shown by a monomeric version of this heterobifunctional ligand **39** was in the millimolar range, similar to the compound *m*-nitrophenyl galactopyranoside (**4**).

**Figure 18:**
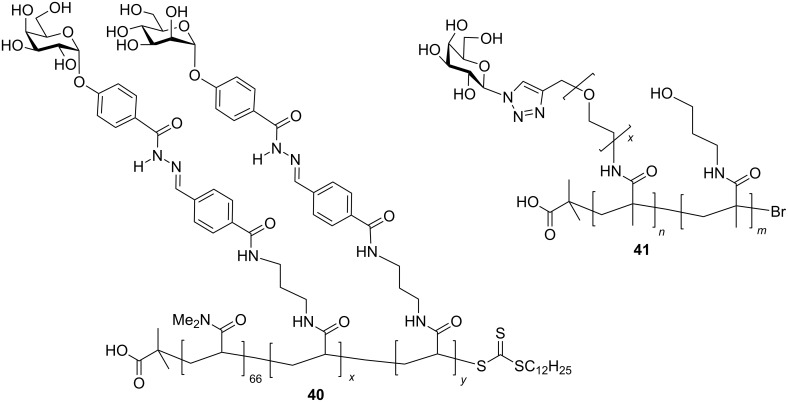
Glycopolymers with exchangeable sugar ligands and variable length linkers.

Fulton and co-workers developed a dynamic combinatorial library of glycopolymers employing exchangeable galactosyl or mannosyl hydrazide functions in conjunction with pendant benzaldehyde groups on the polymer backbone to produce exchangeable hydrazones, e.g., **40** (Figure 18) [69]. They were able to show that in the presence of LTB, the *E. coli* homologue of CTB, the polymer self-optimised its binding affinity for the protein by increasing the proportion of galactosyl residues in the backbone. In the presence of low concentrations of a dihydrazide cross-linking agent, these polymers can also be used to make crosslinked films on surfaces coated with bacterial toxin lectins [72].

Gibson and co-workers made a series of polyacrylates bearing pentafluorophenyl active ester groups which could be subsequently converted to polyacrylamides by reaction with amine linkers of varying lengths [71]. The attachment of galactosyl azides provided a series of glycopolymer inhibitors of CTB (e.g., **41**). They were able to demonstrate that longer linkers between the carbohydrate and polymer backbone gave the best inhibition, probably because they were better able to reach into the binding pocket of the protein.

Multivalent scaffold bearing many galactosyl ligands need not be restricted to organic polymers. Gold nanoparticles coated in galactosyl ligands have been shown to be effective multivalent ligands for cholera toxin [73] and *E. coli* heat-labile toxin [74]. In these cases the objective of the studies was not to invoke inhibition, but rather to exploit the colour changes induced upon crosslinking the gold nanoparticles with CTB or LTB as a strategy for detecting the bacterial toxins.

## Conclusion

Cholera and related diseases caused by other bacterial toxins remain a substantial threat to society. This challenge, and a molecular understanding of the basis of toxin action, has driven the development of diverse inhibitors over many years and this area of research continues to flourish with imaginative and novel strategies emerging for potential antiadhesive therapeutics. Further advances in our understanding of the structural biology of bacterial toxins, in particular the roles of secondary carbohydrate binding sites, will provide new directions for the future development of inhibitors, for example, fucosylated polymers [75], or hybrid inhibitors that can target both the blood group and the GM1 binding pockets. While other emerging, and sophisticated strategies for the use of multivalent scaffolds for displaying (dynamic) libraries of low affinity ligands may accelerate the process of finding effective mimics of the GM1 glycolipid that are simpler in structure and easier to develop into practical therapeutics. Furthermore, the introduction of diverse biophysical methods for studying inhibition mechanisms and novel inhibition assays using intestinal organoids are now providing better quality data and understanding of the action of multivalent inhibitors. The continuous innovation across this field will undoubtedly lead to many more exciting developments for years to come.
